# The Effects of Serum hs-CRP on the Incidence of Lung Cancer in Male Patients with Pulmonary Tuberculosis

**Published:** 2019-07

**Authors:** Youhua JIANG, Kewei NI, Meiyu FANG, Junling LI

**Affiliations:** 1. Department of Thoracic Surgery, Zhejiang Cancer Hospital, Hangzhou 310000, P.R. China; 2. Department of Cardio-Thoracic Surgery, Zhejiang Provincial People’s Hospital, People’s Hospital of Hangzhou Medical College, Hangzhou 310000, P.R. China; 3. Department of Comprehensive Medical Oncology, Zhejiang Cancer Hospital, Hangzhou 310000, P.R. China; 4. State Key Laboratory of Oncology in Southern China, Collaborative Innovation Center for Cancer Medicine, Department of Medical Oncology, Sun Yat-sen University Cancer Center, Guangzhou 510060, P.R. China

**Keywords:** Pulmonary tuberculosis, Serum, High-sensitivity C-reactive protein (hs-CRP), Lung cancer

## Abstract

**Background:**

This study intended to investigate the effects of serum high-sensitivity C-reactive protein (hs-CRP) on the incidence of lung cancer in male patients with pulmonary tuberculosis.

**Methods:**

A total of 1091 male patients with pulmonary tuberculosis in Zhejiang Cancer Hospital, Hangzhou, China from Jan 2009 to Jan 2012 were selected as the research objects. All patients were followed up from the beginning of hospitalization. According to serum hs-CRP level, patients were divided into two groups: group A (hs-CRP < 1 mg/L) and group B (hs-CRP > 3 mg/L). The relationship between baseline hs-CRP and the risk of lung cancer in patients with pulmonary tuberculosis was analyzed by multivariate Cox proportional risk regression model, and the serum levels of hs-CRP between lung cancer patients in all groups and other non-lung cancer patients were compared.

**Results:**

There were differences in age, drinking, smoking, diabetes history, body mass index (BMI), thyroglobulin (TG), history of hypertension and hyperglycemia among the three groups (*P*=0.036, 0.018, 0.040, 0.029, 0.006, 0.034, 0.020, 0.010). The serum levels of hs-CRP in patients with squamous carcinoma, adenocarcinoma and small cell carcinoma were significantly higher than those in non-lung cancer patients (*P*=0.022, 0.043, 0.011). The incidence rates of lung cancer in patients in group B and C were 1.37 and 1.69 times higher than that in group A, respectively.

**Conclusion:**

The increased serum level of hs-CRP will increase the incidence rate of lung cancer in male patients with pulmonary tuberculosis.

## Introduction

Tuberculosis is a chronic disease caused by *Mycobacterium tuberculosis*. It is mainly caused by tuberculosis infection in the lungs, which can invade many organs (1). When the human body is infected with *M. tuberculosis*, the patient’s resistance is reduced or the cell-mediated immunity is elevated, and it will develop tuberculosis. Therefore, the patient clinically needs timely diagnosis and corresponding treatment, to get recovery (2). If the patient is not treated promptly, the patient’s lung will be severely damaged.

The incidence of lung cancer, as the cancer with the highest incidence in China, has been increasing year by year, affecting the health and life of the patients (3). The occurrence of malignant cancer may be closely related to chronic inflammation (4). High-sensitivity C-reactive protein (hs-CRP) is an inflammation-related protein. Hs-CRP will promote the progression of inflammation and induces the expressions of chemokines and adhesion factors.

In this study, the effects of serum hsCRP on the incidence of lung cancer in male patients with pulmonary tuberculosis were explored in order to provide reference for the later clinical treatment.

## Materials and Methods

### Patients

A total of 1091 male patients with pulmonary tuberculosis in our hospital from January 2009 to January 2012 were selected as the research objects. They were aged 18–87 yr old, with an average of 48.32±7.14 yr old. Among them, 24 patients with cancer were aged 32–78 yr, with an average age of 55.39 ±6.48 years. The course of disease was 1–7 yr, with an average of 2.43±0.83 years. Inclusion criteria were: 1) male patients clinically diagnosed with pulmonary tuberculosis; 2) patients without history of cancer; 3) patients who volunteered to participate in this study and signed the informed consent. Exclusion criteria were: 1) patients with severe heart, liver or kidney dysfunction; 2) patients who did not provide blood samples for hs-CRP detection during follow-up; 3) patients who took anticancer drugs before admission; 4) patients with serious mental illness.

The study was approved by the Ethics Committee of Zhejiang Cancer Hospital, and all subjects signed an informed consent form.

The staff who had been trained in our hospital made a face-to-face investigation on the patients’ age, history of drinking, history of smoking, history of diabetes, history of hypertension and so on.

Height and weight were measured barefoot and wearing light clothes. Body mass index (BMI) was calculated as weight (in kg) over height squared (in square meters).

### Detection of serum hs-CRP

Five mL of morning fasting venous blood was collected from the patient and centrifuged at 3000 r/min for 10 min, and the supernatant was taken. The level of hs-CRP in the patient was measured by immunoturbidimetry. All patients were treated with CRP after taking blood samples. According to serum hs-CRP level, patients were divided into two groups: group A (hs-CRP < 1 mg / L) and group B (hs-CRP > 3 mg / L).

### Statistical analysis

The relationship between baseline hs-CRP and the risk of lung cancer in patients with pulmonary tuberculosis was analysed by multivariate Cox proportional risk regression model, and the serum levels of hs-CRP between lung cancer patients in all groups and other non-lung cancer patients were compared.

SPSS 20.0 (Chicago, IL, USA) software package was used for statistical analysis; *t*-test was used for measurement data; *χ*^2^ test was used for enumeration data, and *P*<0.05 suggested that the difference was statistically significant.

## Results

The patients were divided into group A (hs-CRP< 1 mg/L), group B (hs-CRP=1–3 mg/L) and group C (hs-CRP>3 mg/L) according to the serum level of hs-CRP. Baseline data of male patients with pulmonary tuberculosis were statistically significant differences in the age, drinking, smoking, diabetes history, BMI, thyroglobulin (TG), history of hypertension and hyperglycaemia among the three groups (*P*=0.036, 0.018, 0.040, 0.029, 0.006, 0.034, 0.020, 0.010) (Table 1). A total of 24 out of 1091 male patients with pulmonary tuberculosis had lung cancer, and the serum levels of hs-CRP in patients with squamous carcinoma, adenocarcinoma and small cell carcinoma were significantly higher than those in non-lung cancer patients, and the differences were statistically significant (*P*=0.022, 0.043, 0.011) (Table 2).

**Table 1:** Baseline data of male patients with pulmonary tuberculosis [n(%)]

***Variable***	***n***	***Group A***	***Group B***	***Group C***	***x^2^***	***P***
Age (yr)					5.421	0.036
≤50	522	325(62.26)	132(25.28)	65(12.46)		
>51	569	288(50.61)	165(28.99)	116(20.40)		
Drinking					7.276	0.018
No	233	127(54.50)	64(27.46)	42(18.04)		
Yes	858	386(44.98)	283(32.98)	189(22.04)		
Smoking					4.997	0.040
No	377	171(45.35)	127(33.68)	79(20.97)		
Yes	714	307(42.99)	251(35.15)	156(21.86)		
Diabetes history					6.158	0.029
No	989	569(57.53)	262(26.49)	158(15.98)		
Yes	102	44(43.13)	34(33.33)	24(23.54)		
BMI (kg/m^2^)					8.531	0.006
<24	411	266(64.72)	85(20.68)	60(14.60)		
24–28	469	257(54.79)	135(28.78)	77(16.43)		
≥28	211	89(42.18)	77(36.49)	45(21.33)		
TG>1.7 mmol/L					5.629	0.034
No	973	574(58.99)	266(27.33)	133(13.68)		
Yes	118	49(41.52)	38(32.20)	31(26.28)		
History of hypertension					7.037	0.020
No	856	481(56.19)	253(29.55)	122(14.26)		
Yes	235	91(38.72)	89(37.87)	55(23.41)		
History of hyperglycemia					8.158	0.010
No	879	504(57.33)	244(27.75)	131(14.92)		
Yes	212	102(48.11)	61(28.77)	49(23.12)		

**Table 2:** Comparisons of serum levels of hs-CRP between patients with lung cancer in each group and patients with non-lung cancer (mg/L)

***Pathological type***	***n***	***Level of hs-CRP***	***t***	***P***
Squamous carcinoma	14	9.86±4.62	2.504	0.022
Adenocarcinoma	6	9.09±4.53	2.059	0.043
Small cell carcinoma	4	8.73±5.33	2.573	0.011
Non lung cancer group	1067	0.73±0.45		

Patients in group A (hs-CRP<1 mg/L) were used as control group, and the results of multivariate Cox proportional risk regression model analysis showed that the incidence rates of lung cancer in patients in group B and C were 1.37 and 1.69 times as high as that in patients in group A, respectively (Table 3).

**Table 3:** Multivariate Cox proportional risk regression model analysis

***Level of hs-CRP***	***Beta***	***Wald***	***HR***	***P***	***95% CI***
<1 mg/L			1.00		
1–3 mg/L	0.552	5.047	1.737	0.024	2.397–4.184
>3 mg/L	1.259	5.417	3.522	0.019	2.025–3.987

## Discussion

Lung cancer is one of the most common cancer in the world, and there are about 1.38 million new cases of lung cancer each year. The mortality rate of lung cancer patients in China is second only to that of gastric cancer and liver cancer, which poses a serious threat to human health and life (5). Hs-CRP is an acute phase protein released during human inflammatory response. It is an important indicator of the diagnosis and prognosis of diseases such as tissue injury and clinical infection in clinic (6). When the cancer occurs, the hs-CRP in the body will rise abnormally to 10–100 times the normal value and it will inhibit the process of cell apoptosis, stimulate angiogenesis, increase vascular permeability and promote tumor progression (7, 8). Patients with pulmonary tuberculosis are prone to lung cancer because of the long-term illness, severe lung injury, and the effects of inflammatory cytokines (9). In this study, the effects of serum hs-CRP on the incidence of lung cancer in male patients with pulmonary tuberculosis were explored in order to provide reference for the later clinical treatment.

The CRP level is measured by routine methods in clinical laboratories. The range of CRP is usually 3–5 mg/L, and the sensitivity is low. The measured range of CRP cannot meet the clinical needs, and it inhibits the use of CRP in clinical diagnosis and prediction (10). However, in recent years, the level of CRP has been measured by latex enhanced immunoturbidimetry, which can improve the sensitivity. The measured range of CRP is 0.15–10 mg/L, and the accuracy is high. The measured CRP is called hs-CRP (11). The results of this study showed that there were differences in the age, drinking, smoking, diabetes history, BMI, TG, history of hypertension and hyperglycemia among the three groups, and the differences were statistically significant (*P*<0.05). This is similar to the results of previous studies (12). The age, smoking, drinking, BMI and other factors were corrected in this study with patients in group A (hs-CRP<1 mg/L) as control group, and the multivariate Cox proportional risk regression model analysis results showed that the incidence rates of lung cancer in patients in group B and C were 1.37 and 1.69 times as high as that in group A respectively. The biological mechanism that low-grade chronic inflammation increases the incidence of lung cancer in male patients with pulmonary tuberculosis is not completely clear. However, when the patient’s body is in chronic low-grade inflammatory state, inflammatory cells will secrete interleukin-1 (IL-1), interleukin-6 (IL-6) and TNF-α to induce hepatic secretion of hs-CRP. Hs-CRP will activate smooth muscle cells, endothelial cells and mononuclear cells, and then promote the development of inflammation and the expressions of adhesion factors and chemokines to induce cell carcinogenesis. The oxidation in the process of persistent low-grade chronic inflammation can cause damage to cells, lead to mutations in the function of proteins involved in gene repair, mutations in tumor suppressor genes and induction of pulmonary cell carcinogenesis (13). At the same time, chronic inflammation will also activate inflammatory cytokine infiltration and humoral immunity in patients, increase vascular permeability, and promote the formation of blood vessels and lung tumor progression (14). The results of this study showed that there is a certain correlation between the level of hs-CRP in lung cancer patients and the pathological type of lung cancer, and some studies have indicated that level of hs-CRP is gradually increased with the increase of clinical stages in patients with lung cancer (15).

## Conclusion

The elevated serum level of hs-CRP will increase the incidence rate of lung cancer in male patients with pulmonary tuberculosis. It suggests that the detection of serum tumor markers in clinic should be combined with the detection of serum hs-CRP level. The hs-CRP level in serum of patients is used as the basis to provide a sufficient basis for the diagnosis of lung cancer patients, so that the prognosis of the patients is accurately judged and timely treatment is given.

## Ethical considerations

Ethical issues (Including plagiarism, informed consent, misconduct, data fabrication and/or falsification, double publication and/or submission, redundancy, etc.) have been completely observed by the authors.
